# Is Shaving Favorable to Health?—A Plea for Beards

**Published:** 1862-12

**Authors:** A. Mercer Adams


### IS SHAVING FAVORABLE TO HEALTH?—A PLEA FOR BEARDS.

BY A. MERCER ADAMS, M.D.

Late Physician to the Dumfries and Galloway Royal Infirmary.

Dr. Adams holds that shaving is neither warranted by antiquity, necessary for comfort, nor conductive to health; and his arguments have much to recommend them. He reminds us, also, that the prevalence of shaving in this and the last century originated in a miserable piece of sycophantism toward two French monarchs, (Louis XIII. and Louis XIV.,) who ascended the throne during their minority, the courtiers making their chins bare in compliment to their beardless princes, and that shaving, in fact, is nothing more than a perverse fashion. And, certainly, there is something to be said in favor of the hygienic virtues of the beard. For example—

The beard seems as truly designed by nature to aid in the preservation of the health as is the hair covering the cranium. The moustache is emphatically nature's simple respirator, while the hair covering the jaws and throat is intended to afford warmth and protection to the delicate structures in the vicinity, especially the fauces and the larynx. In shaving, then, do we not destroy the provisions which have been made for the maintenance of the health? We are only aware of one author who has directed special attention to this inquiry, and who has furnished us with any statistics bearing on the subject. Dr. Szokalski has given us the details of his observations, made, in 1853, on fifty-three strong, healthy men, whose ages ranged from twenty-five to forty-five, who shaved the face, after having previously worn the whole beard. All of them at first experienced very unpleasant sensations of cold, but only fourteen of them became speedily accustomed to the change, and experienced no further inconvenience. The others suffered more or less, in various ways. Twenty-seven had painful affections of the teeth and jaws—eleven having toothache and facial neuralgia, and sixteen rheumatism of the gums, with and without abscesses. In six cases there was obstinate enlargement of the submaxillary glands; and in thirteen there was a rapid increase of the caries in previously affected teeth, requiring extraction of the aching grinders. He compared the statistics of toothache in thirty men, of the age of thirty years, one-half of whom wore the beard, and the others shaved. Among those of the first class there had been only eight teeth extracted, while among the others there had been no less than twenty-six extractions. All the cases of dental neuralgia which came under his notice, as the results of shaving, were obstinate and tedious in their character; in a few the disease assumed an intermittent type; and in two cases all remedies proved unavailing, until the beard had been allowed to grow once more. He is therefore firmly of opinion that the growth of the beard is conducive to health, and that shaving renders weakly persons more susceptible of violent alterations of temperature, and consequently more liable to disease.

The beard also acts beneficially as a respirator, for it not only mechanically prevents the entrance of foreign particles into the air passages, but it also lessens the coldness of the air we breathe, by imparting to it, as it passes through the thick moustache, some of the heat which has been left there by the warm breath just expired. The utility of the beard, as a hygienic agent, was recognized many years ago by one of the wisest physicians and most benevolent men of modern times, the late Professor Alison, of Edinburgh. The stone hewers in the neighborhood of Edinburgh are known to be peculiarly short-lived—few of them reaching more than forty years—on account of the prevalence of phthisis, caused by their constantly breathing the fine silicious particles which fill the air as they are working the freestone. To prevent the inhalation of these irritating bodies, Dr. Alison recommended these stonemasons to allow the beard to grow on the upper lip. (The mason, as the reader may have observed, generally keeps his mouth closed in hewing stones, and breathes by the nostrils alone—in fact we all breathe more by the nose than by the mouth.) The moustache, therefore, became very generally worn among that class of men, and was found very efficacious in arresting the fine dust, which must otherwise have entered the lungs.

The beard and moustache began to be worn a few years ago—before the fashion became so universal—by railway guards, engine drivers, etc., for similar reasons. These men are exposed to many vicissitudes of temperature, and are also constantly obliged to inspire air loaded with minute particles of dust, ashes and carbonaceous matter. The beard is of great service to them, by rendering them less susceptible of violent alternations of temperature, and by preventing the inhalation of the deleterious particles in the air. I have made many careful inquiries among the officials of the Great Northern Railway (and from being medical officer to the large body of *employés* in the works at Boston, and to the engine-drivers, etc., over a large portion of the line, I have had peculiar facilities for making such an investigation) as to whether the men who wore the beard enjoyed a greater immunity from disease than those who shaved. The result of all my inquiries and personal observations has been decidedly in favor of the beard as a protection to the health. Pulmonary and respiratory affections are comparatively rare among the bearded railway officials; and all those whom I questioned on the subject assured me that they found the beard an indiscribable comfort, and were quite convinced of its protective virtues.

By the courtesy of Mr. Seymour Clarke, the accomplished manager of the Great Northern Railway, I am able to give some official information respecting the influence of the beard on the health of the servants on that extensive line. Mr. Clarke informs us, in a letter dated 2d November, that only sixteen enginemen and firemen shave the beard; that seventy-seven let the beard grow, and forty-two cultivate both the beard and moustache. "The prevailing opinion among the men," says Mr. Clarke, "is that those who wear the beard and moustache enjoy better health than those who shave."—*Edinb. Med. Journal*, December, 1861; *Half-Yearly Abstr. of Med. Sciences.*

